# Association of *MBL2* gene polymorphisms and MBL levels with dilated cardiomyopathy in a Chinese Han population

**DOI:** 10.1186/s12920-023-01787-2

**Published:** 2024-01-02

**Authors:** Yujie Mao, Hong Wei, Yugang Gong, Lei Peng, Yu Chen

**Affiliations:** 1 Institute of Dermatology and Venereology, Sichuan Provincial People’s Hospital, University of Electronic Science and Technology of China, Chengdu, 610072 China; 2 Department of Ophthalmology and Vision Research Laboratory, West China Hospital, Sichuan University, Chengdu, China; 3 Department of Nephrology, Sichuan Provincial People’s Hospital, University of Electronic Science and Technology of China, Chengdu, 610072 China; 4 Department of Cardiology, Sichuan Provincial People’s Hospital, University of Electronic Science and Technology of China, Chengdu, 610072 China

**Keywords:** Dilated cardiomyopathy, Mannose-binding lectin, Polymorphism, Gene, Risk

## Abstract

**Background:**

It has been reported that *Mannose-binding lectin 2 (MBL2)* gene polymorphisms and expression levels are related to dilated cardiomyopathy (DCM). This study aimed to investigate the potential association between *MBL2* gene polymorphisms and the pathogenesis of DCM.

**Methods:**

Five single nucleotide polymorphisms (SNPs) of the *MBL2* gene were genotyped in 440 DCM patients and 532 controls in Southwest China. A luciferase reporter assay was used to detect the transcriptional activity the different genotypes. MBL serum levels, left ventricle ejection fraction (LVEF) and lower left ventricular end-diastolic diameter (LVEDD) were measured.

**Results:**

The rs11003125 C allele increased the transcriptional activity of the *MBL2* promoter compared with the rs11003125 G allele. The rs11003125 CC carriers had higher MBL serum levels, LVEF and LVEDD than the rs11003125 CG and GG carriers.

**Conclusions:**

Our study first revealed that *MBL2* polymorphisms and serum MBL levels were associated with DCM. Allele C in rs11003125 of *MBL2* may upregulate the expression levels of MBL. High serum MBL levels may be a protective factor in DCM pathogenesis.

## Introduction

Dilated cardiomyopathy (DCM) is a structural heart disease characterized by dilation and impaired contraction of the left ventricle or both ventricles, with a prevalence of up to 1:2500 in the general population [1]. It has been demonstrated that DCM is the third most common cause of heart failure and the most common condition requiring heart transplantation [2–4]. At present, the etiology of DCM is unclear [5]. Some evidence has shown that the occurrence of DCM is exacerbated by factors such as viral infection, inflammation, autoimmune diseases, toxic and metabolic damage, heredity, myocardial ischemia and tachycardia [5–9].

Mannose-binding lectin (MBL) is a protein found in plasma and is part of the innate immune system that can activate the complement cascade [10]. It is typically involved in antimicrobial and proinflammatory functions [11]. The level of the human MBL protein and its functionality are regulated mainly by single nucleotide polymorphisms (SNPs) of the *MBL2* gene, which is located at chromosomal region 10q11.2-q21. The SNPs rs10082466, rs2120132, rs2099902, rs7096206 and rs11003125 in the *MBL2* gene have been proven to be functionally important and deleterious to its structure and expression [12–15].

Some studies have shown that *MBL2* SNPs in promoter and exonic regions regulate MBL serum levels in various autoimmune and infectious diseases [16–19]. MBL and the lectin complement pathway play important roles in vascular dysfunction, and cardiomyopathy has also been reported [20]. Considering that DCM is associated with factors such as myocardial inflammation, infection and immune responses and that MBL is related to these factors, we hypothesized that *MBL2* polymorphisms and circulating MBL levels are associated with the occurrence of DCM. To test our hypothesis, a case‒control study was performed to evaluate polymorphisms of the *MBL2* gene and serum MBL levels in DCM patients and healthy controls from a Chinese Han population living in Southwest China.

## Materials and methods

### Study subjects

This case‒control study enrolled 440 unrelated DCM patients from Sichuan Provincial People’s Hospital between January 2016 and December 2020. The clinical diagnosis was performed according to the revised criteria established by the 1995 WHO/International Society and Federation of Cardiology Task Force on the Classification of Cardiomyopathy (DCM group) [21]. The control group consisted of 532 subjects from a routine health survey. All subjects were Han Chinese living in Sichuan Province in southwest China. Patients with a history of hypertension, congenital heart disease, coronary heart disease, cardiac valve disease, tachyarrhythmia, heavy alcohol intake, acute viral myocarditis, systemic diseases of putative autoimmune origin or skeletal myopathies were intentionally excluded. The healthy controls also had no evidence of any organic cardiac diseases, hypertension, viral myocarditis, autoimmune diseases, heavy alcohol intake or skeletal myopathies. Echocardiography was performed to evaluate the left ventricular dimensions (left ventricular end-diastolic diameter, LVEDD) and left ventricular ejection fraction (LVEF) in all the subjects. The study protocol was reviewed and approved by the Ethics Committee of Sichuan Provincial People’s Hospital (No. 16,027), and all the subjects provided informed consent.

### SNP selection and genotyping

Five SNPs (rs10082466, rs2120132, rs2099902, rs7096206, and rs11003125) were selected for our analysis according to a previous comprehensive study of the human *MBL2* gene using in silico analysis [12].

EDTA-treated peripheral blood samples from all enrolled subjects were collected for genotyping. Genomic DNA was extracted from leukocytes by an extraction kit according to the manufacturer’s instructions (Bioteke Corporation, Beijing, China). The SNP genotyping work was performed using a custom-by design 48-Plex SNPscanTM Kit (Genesky Biotechnologies Inc., Shanghai, China) as previously described [22]. This technique was based on double ligation and multiplex fluorescence PCR. For quality control, approximately 4% of all samples were randomly selected for Sanger sequencing, and the results were 100% consistent.

### Dual-luciferase reporter assay

A dual-luciferase reporter assay was used to detect the transcriptional activity of the different genotypes. The protocol has been described previously in detail [23]. A 748 bp DNA fragment in the *MBL2* promoter containing the rs11003125 C or G genotype was synthesized by Sangon Corporation, inserted into the pGL3-basic vector and confirmed by Sanger sequencing. Five hundred nanograms of the pRL-TK vector and 500 ng of the pGL3-basic vector, pGL3-rs11003125 C vector or pGL3-rs11003125 G vector were transfected into human embryonic kidney 293 cells by Invitrogen Lipofectamine 3000 (Invitrogen, Waltham, Massachusetts, USA). Luciferase activity was measured at 48 h after transfection using the Promega Dual-Luciferase Reporter Assay System (Promega, Madison, Wisconsin, USA). The construction of each vector was tested in triplicate. The luciferase activity was computed with pGL3/pRL-TK.

### Measurement of serum MBL levels

Serum MBL was obtained from the venous blood of 440 DCM patients and 532 healthy controls. The MBL concentration was measured with an enzyme-linked immunosorbent assay (ELISA) (Human MBL DuoSet, RD Systems, USA). All the measurements were performed in duplicate. Serum MBL levels were determined by a standard curve using mean optical density values.

### Statistical analysis

Data analyses were conducted with SPSS 26.0 statistical software (SPSS, Inc., Chicago, IL, USA). The normality of continuous data was evaluated using the Shapiro‒Wilk and Kolmogorov‒Smirnov tests. Normally distributed data are expressed as the mean ± SD, and Student’s t test was applied. Nonnormally distributed data are expressed as the median and interquartile range (IQR), and the Kruskal‒Wallis test was applied. Pearson’s chi-square test was used for categorical variables in the comparison of Hardy-Weinberg equilibrium and allelic and genotypic frequencies in different groups. The Hardy-Weinberg equilibrium was tested for the patients and controls. SNPstats has been used to calculate genotypic associations in a case‒control pattern, assuming codominant, dominant, recessive, or overdominant genetic models [24]. Odds ratios (ORs) and 95% confidence intervals (CIs) were reported to evaluate the effects of any difference in allelic and genotypic distributions. A Bonferroni-corrected *p*-value of 0.01 was set to control for Type I errors. When the *p*-value was < 0.01, the differences in allele and genotype distributions were regarded as statistically significant between DCM patients and control subjects. When the *p*-value was less than 0.05 in other tests, the difference was regarded as statistically significant.

## Results

### Clinical data

**Fig. 1:**
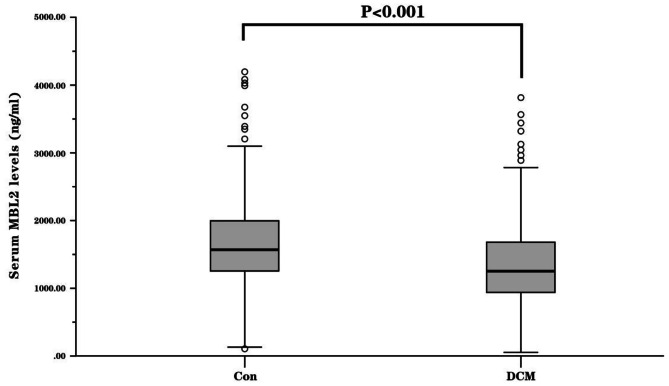
Distribution of serum MBL levels in DCM patients and controls

The baseline characteristics of the DCM patients and controls are presented in Table 1. There were no significant differences between DCM patients and healthy controls in age and sex. DCM patients had statistically lower systolic blood pressure (SBP), diastolic blood pressure (DBP), and LVEF than the controls. Conversely, the LVEDD, heart rate and brain natriuretic peptide (BNP) level in patients with DCM were significantly higher than those in the control group. Serum MBL2 levels in DCM patients were much lower than those in controls (Fig. 1).

**Table 1:** Baseline characteristics of DCM patients and controls

Variables	DCM (n = 440)	Controls (n = 532)
Age (years)	54.50 ± 8.60	55.41 ± 9.30
Gender (male/female)	273/167	309/223
SBP (mmHg)	103.19 ± 8.40	117.85 ± 10.49*
DBP (mmHg)	62.35 ± 9.12	71.92 ± 5.68*
LVEF (%)	32.98 ± 6.19	62.77 ± 10.41*
LVEDD (mm)	62.56 ± 5.24	45.78 ± 5.04*
Heart rate (beats/min)	79.80 ± 12.34	73.02 ± 11.36*
BNP (pg/mL)	3635.79 ± 1481.74	84.12 ± 32.97*
MBL (ng/ml)	1306.00(937.50–1676.00)	1570.50(1245.25-1999.50)*

The data are presented as the mean value ± SD or number (%) of subjects. *, *P* < 0.05, comparison between controls and DCM patients. DCM, dilated cardiomyopathy; SBP, systolic blood pressure; DBP, diastolic blood pressure; LVEF, left ventricular ejection fraction; LVEDD, left ventricular end-diastolic diameter; BNP, brain natriuretic peptide

### Association between MBL2 polymorphisms and the risk of DCM

The genotype and allelic frequencies of the five SNPs (rs10082466, rs2120132, rs2099902, rs7096206, and rs11003125) between DCM patients and healthy controls are shown in Table 2. The rs10082466, rs2120132, rs2099902, rs7096206, and rs11003125 SNPs in DCM patients and control subjects were in Hardy-Weinberg equilibrium (*p* > 0.05). The *p*-values were 0.77 for rs10082466, 1.00 for rs2120132, 0.50 for rs2099902, 0.64 for rs7096206, and 0.07 for rs11003125, respectively. Significant differences in genotypes and allelic frequencies were found at rs11003125, indicating that this SNP might play an important role in the pathogenesis of DCM. As shown in Table 2, the rs11003125 polymorphism was associated with a predisposition to DCM according to the codominant (*P* = 0.007) and dominant (*P* = 0.002) models. The significant association between rs11003125 and DCM risk was confirmed by Bonferroni correction (corrected *P* = 0.01).

**Table 2:** Allele and genotype distributions of *MBL2* genetic polymorphisms in DCM patients and controls

Model	Genotype	DCM, n = 440 (%)	Controls, n = 532 (%)	OR (95% CI)	*P* value
**rs10082466**					
Codominant	A/A	293 (66.6%)	354 (66.5%)	1.00	
	G/A	133 (30.2%)	162 (30.4%)	0.99 (0.75–1.31)	0.986
	G/G	14 (3.2%)	16 (3.0%)	1.06 (0.51–2.20)	
Dominant	A/A	293 (66.6%)	354 (66.5%)	1.00	0.987
	G/A- G/G	147 (33.4%)	178 (33.5%)	1.00 (0.76–1.30)	
Recessive	A/A- G/A	426 (96.8%)	516 (97.0%)	1.00	0.876
	G/G	14 (3.2%)	16 (3.0%)	1.06 (0.51–2.20)	
Overdominant	A/A- G/G	307 (69.8%)	370 (69.5%)	1.00	0.940
	G/A	133 (30.2%)	162 (30.4%)	0.99 (0.75–1.30)	
Allele	A	719 (82%)	870 (82%)	1.00	0.972
	G	161(18%)	194 (18%)	1.00 (0.79–1.26)	
**rs2120132**					
Codominant	T/T	290 (65.9%)	364 (68.4%)	1.00	
	C/T	136 (30.9%)	153 (28.8%)	0.90 (0.68–1.18)	0.703
	C/C	14 (3.2%)	15 (2.8%)	0.85 (0.41–1.80)	
Dominant	T/T	290 (65.9%)	364 (68.4%)	1.00	0.406
	C/T- C/C	150 (34.1%)	168 (31.6%)	0.89 (0.68–1.17)	
Recessive	T/T- C/T	426 (96.8%)	517 (97.2%)	1.00	0.741
	C/C	14 (3.2%)	15 (2.8%)	0.88 (0.42–1.85)	
Overdominant	T/T- C/C	304 (69.1%)	379 (71.2%)	1.00	0.465
	C/T	136(30.9%)	153 (28.8%)	0.90 (0.68–1.19)	
Allele	T	716 (81%)	881 (83%)	1.00	0.410
	C	164(19%)	183 (17%)	0.91 (0.72–1.14)	
**rs2099902**					
Codominant	T/T	235 (53.4%)	289 (54.3%)	1.00	
	C/T	176 (40.0%)	211 (39.7%)	1.03 (0.79–1.34)	0.918
	C/C	29 (6.6%)	32 (6.0%)	1.11 (0.66–1.90)	
Dominant	T/T	235 (53.4%)	289 (54.3%)	1.00	0.776
	C/T- C/C	205 (46.6%)	243 (45.7%)	1.04 (0.81–1.34)	
Recessive	T/T- C/T	411 (93.4%)	500 (94%)	1.00	0.713
	C/C	29 (6.6%)	32 (6.0%)	1.10 (0.66–1.85)	
Overdominant	T/T- C/C	264 (60.0%)	321 (60.3%)	1.00	0.915
	C/T	176 (40.0%)	211 (39.7%)	1.01 (0.78–1.31)	
Allele	T	646 (73%)	789 (74%)	1.00	0.710
	C	234 (27%)	275 (26%)	0.96 (0.79–1.18)	
**rs7096206**					
Codominant	C/C	291 (66.1%)	373 (70.1%)	1.00	
	C/G	136 (30.9%)	143 (26.9%)	1.22 (0.92–1.61)	0.383
	G/G	13 (3.0%)	16 (3.0%)	1.04 (0.49–2.20)	
Dominant	C/C	291 (66.1%)	373 (70.1%)	1.00	0.185
	C/G- G/G	149 (33.9%)	159 (29.9%)	1.20 (0.92–1.58)	
Recessive	C/C- C/G	427 (97.0%)	516 (97.0%)	1.00	0.961
	G/G	13 (3.0%)	16 (3.0%)	0.98 (0.47–2.06)	
Overdominant	C/C- G/G	304 (69.1%)	389 (73.1%)	1.00	0.167
	C/G	136 (30.9%)	143 (26.9%)	1.22 (0.92–1.61)	
Allele	C	718 (82%)	889 (84%)	1.00	0.255
	G	162 (18%)	175(16%)	0.87 (0.69–1.10)	
**rs11003125**					
Codominant	G/G	133 (30.2%)	115 (21.6%)	1.00	
	C/G	220 (50.0%)	288 (54.1%)	0.66 (0.49–0.90)	0.007*
	C/C	87 (19.8%)	129 (24.2%)	0.58 (0.40–0.84)	
Dominant	G/G	133 (30.2%)	115 (21.6%)	1.00	0.002*
	C/G- C/C	307 (69.8%)	417 (78.4%)	0.64 (0.48–0.85)	
Recessive	G/G- C/G	353 (80.2%)	403 (75.8%)	1.00	0.094
	C/C	87 (19.8%)	129 (24.2%)	0.77 (0.57–1.05)	
Overdominant	G/G- C/C	220 (50.0%)	244 (45.9%)	1.00	0.199
	C/G	220 (50.0%)	288 (54.1%)	0.85 (0.66–1.09)	
Allele	G	486 (55%)	518 (49%)	1.00	0.003*
	C	394 (45%)	546 (51%)	0.76 (0.63–0.91)	

*MBL2*, mannose-binding lectin 2; DCM, dilated cardiomyopathy; CI, confidence interval; OR, odds ratio; *, *P* value < 0.05, comparison between controls and DCM patients

**Fig. 2:**
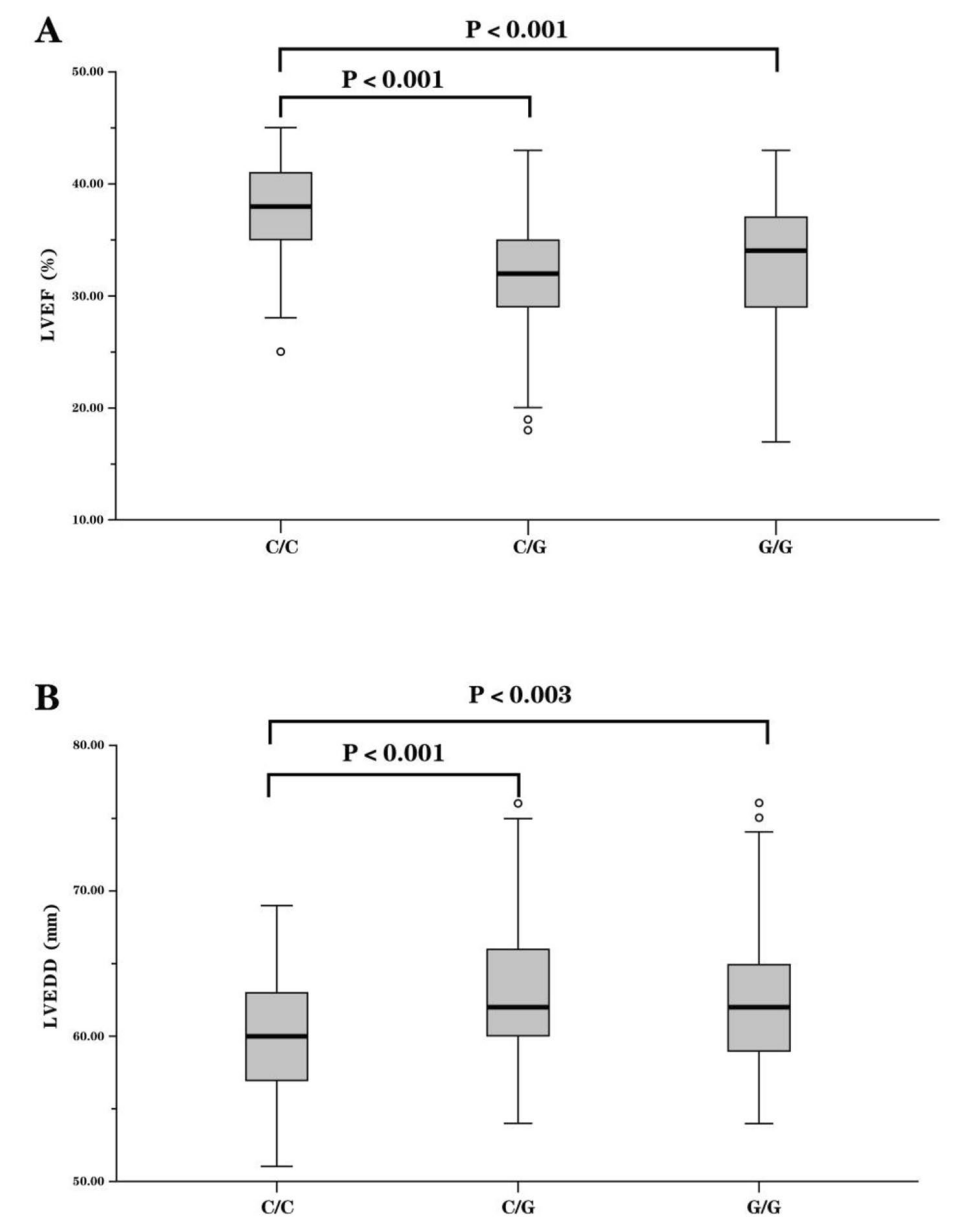
Distribution of LVEF (**A**) and LVEDD (**B**) in DCM patients stratified by rs11003125 genotype. □, interquartile ranges (IQRs).

Subjects with the CC genotype and C allele had a significantly lower risk of DCM than those with the GG genotype and G allele (CC versus GG, OR = 0.58, 95% CI = 0.40–0.84; C versus G, OR = 0.76, 95% CI = 0.63–0.91, *P* = 0.003). There was no significant association between rs10082466, rs7096206 or rs2099902 and DCM risk. Further analysis of the correlation between the rs11003125 genotype and clinical parameters determined via echocardiography was performed in our study. As shown in Fig. 2, the rs11003125 CC carriers had significantly higher LVEF than the rs11003125 CG (*P* < 0.001) and GG carriers (*P* < 0.001), and the rs11003125 CC carriers exhibited lower LVEDD than the rs1003125 CG (*P* < 0.001) and GG carriers (*P* = 0.003).

### The rs11003125 C allele increased transcriptional activity

**Fig. 3:**
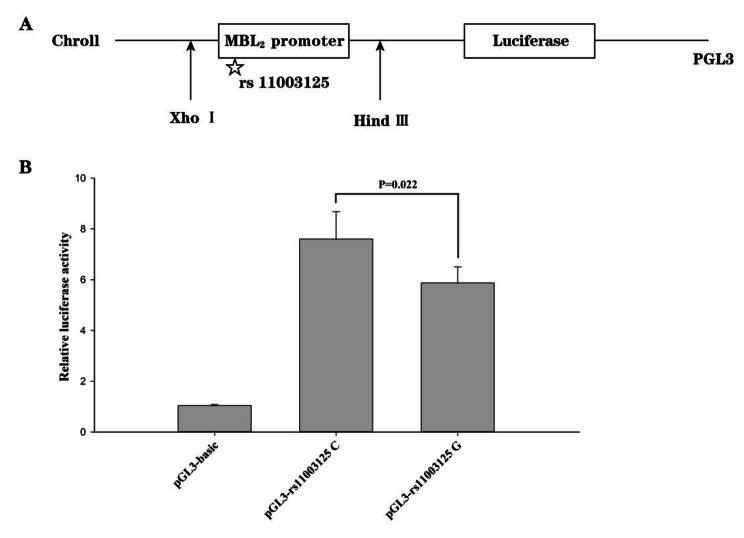
The rs11003125 C allele increased the transcriptional activity of the *MBL2* promoter. **A**, Schematic representation of the constructed pGL3- rs11003125 C/G vector. **B**, The constructed vectors were transfected into human embryonic kidney 293 cells and luciferase activity was measured 48 h after transfection (*P* < 0.05)

Plasmids containing the rs11003125 C or G allele were constructed, and reporter activity was measured to verify the influence of the rs11003125 polymorphism on the transcriptional activity of the *MBL2* promoter. A schematic representation of the plasmid construction is shown in Fig. 3A. The pGL3-rs11003125 C and G vectors exhibited higher luciferase activity than the empty vector (Fig. 3B). The transcriptional activity of the *MBL2* promoter was increased significantly by the pGL3-rs11003125 C vector compared with the pGL3-rs11003125 G vector (*P* = 0.022).

### The rs11003125 CC genotype was associated with increased serum MBL levels

**Fig. 4:**
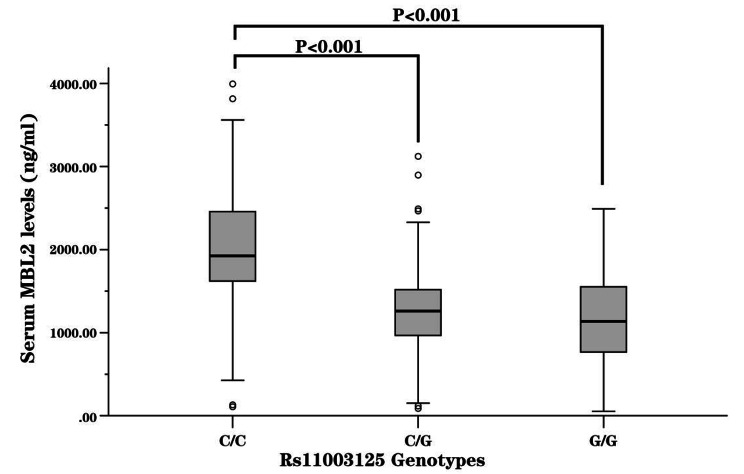
Distribution of serum MBL levels in DCM patients stratified by rs11003125 genotype. □, interquartile ranges (IQRs).

To determine whether rs11003125 influenced MBL expression, we analyzed the serum MBL levels in DCM patients who were stratified by rs11003125 genotype. The results revealed that the median MBL levels of the rs11003125 CC, CG and GG carriers were 1924.00 ng/ml (IQRs: 1611.00-2462.00), 1263.00 ng/ml (IQRs: 962.25-1518.25) and 1132.00 ng/ml (IQRs: 757.50–1554.00), respectively. The rs11003125 CC carriers had significantly increased serum MBL levels compared to the rs11003125 CG (*P* < 0.001) and GG carriers (*P* < 0.001) (Fig. 4).

## Discussion

This study provides the first evidence that a polymorphism in the *MBL2* gene (rs11003125) is significantly associated with Chinese Han DCM patients living in Southwest China. The expression levels of MBL were upregulated in DCM patients carrying the rs11003125 CC genotype compared with those carrying the rs11003125 GG or GC genotype. Moreover, the rs11003125 C allele increased the transcriptional activity of the *MBL2* promoter in contrast with the rs11003125 G allele. The results showed that the rs11003125 CC genotype and C allele are strongly associated with a reduced risk of DCM. In other words, the rs11003125 GG genotype and G allele significantly elevated the risk of DCM.

The pathogenesis of DCM is not fully understood. Infection, inflammation and autoimmunity are involved in the progression of the disease. Disturbance of the immune system is often reported to be associated with DCM. Myocardial inflammation is one of the most common mechanisms in DCM [25]. MBL deficiency is related to susceptibility to autoimmune diseases, and serum MBL levels vary remarkably due to the variant alleles in the *MBL2* gene [26]. Previous studies have shown that the absence of MBL affects the occurrence of cardiovascular complications and myocardial ischemia [27, 28]. Similarly, downregulation of MBL in DCM was found in our study, suggesting that MBL may be involved in the pathogenesis of DCM. However, not all individuals with downregulated MBLs suffer from DCM. This phenomenon suggested that gene polymorphisms might play a role in these processes.

The influence of *MBL2* gene polymorphisms on human diseases has been reported in many studies. Kim SH et al. investigated three SNPs (rs11003125, rs11003124, rs7096206) in the *MBL2* promoter and found that polymorphisms of the *MBL2* gene increased susceptibility to the development of diisocyanate-induced occupational asthma [29]. Mokhtari MJ et al. demonstrated that *MBL2* gene polymorphisms were associated with dental caries in Iranian adults and that the GG and GC genotypes of the *MBL2* rs11003125 polymorphism remarkably increased caries risk according to the dominant model [30]. Zhang N et al. showed that the rs1800450 and rs11003125 SNPs of the *MBL2* gene had strong linkage disequilibrium and were associated with type 2 diabetes in the North Chinese Han population [14]. Subjects with the CC genotype of rs11003125 had much higher serum MBL levels. Moreover, Yokoyama E and colleagues proved that the *MBL2* rs11003125 GG or GC genotype was significantly associated with patients with cystic fibrosis as a risk factor [31]. Our study showed that the rs11003125 CC genotype and C allele of *MBL2* were associated with a decreased risk of DCM, suggesting that G increases susceptibility while C offers protection. The results were consistent with the above previous studies.

According to the literature, rs11003125 located in the *MBL2* promoter and SNPs in the promoter of a gene were supposed to influence its expression level [32, 33]. Thus, we investigated the relationship of rs11003125 with the expression levels of MBL. The results showed that the rs11003125 CC genotype was related to higher expression levels of MBL than the GG and GC genotypes. Analogously, MBL levels of the rs11003125 CC group in diabetic nephropathy [14], sepsis and common infectious diseases were also found to be significantly higher [34, 35]. The results of a luciferase reporter assay suggested that the rs11003125 C allele increased the transcriptional activity of the *MBL2* promoter. Previous in silico analysis revealed that rs11003125 is located at the binding site of the transcription factor in the *MBL2* gene [12]. The change from allele G to allele C might increase the motif score or the binding efficiency between transcription factors and the binding site. Our findings implied that the rs11003125 C allele increased the transcriptional activity of the *MBL2* promoter by upregulating the binding efficiency between transcription factors and the binding site. This study provided evidence that CC carriers with DCM at the rs11003125 polymorphism were related to two important clinical parameters of cardiac function, LVEF and LVEDD. DCM patients with the rs11003125 CC genotype had much higher LVEF and lower LVEDD than the other genotype carriers. This result revealed that genotypic variations might be prognostic factors for DCM. The above evidence suggested that rs11003125 might play a protective role in the development of DCM by increasing the transcriptional activity of the *MBL2* promoter and MBL expression.

## Conclusion

Our findings demonstrated that *MBL2* polymorphisms and circulating MBL levels are associated with the occurrence of DCM. High serum MBL levels may be a protective factor in DCM pathogenesis, and the C allele of *MBL2* rs11003125 may upregulate the expression of MBL by increasing the transcriptional activity of the *MBL2* promoter.

## Data Availability

The datasets used and/or analyzed during the current study are available from https://weibo.com/u/5672759868?tabtype=album&uid=5672759868&index=0.
